# Technical assessment of small-scale wind power for residential use in Mexico: A Bayesian intelligence approach

**DOI:** 10.1371/journal.pone.0230122

**Published:** 2020-03-12

**Authors:** Monica Borunda, Javier de la Cruz, Raul Garduno-Ramirez, Ann Nicholson

**Affiliations:** 1 Conacyt–Instituto Nacional de Electricidad y Energías Limpias, Cuernavaca, Mexico; 2 Instituto Nacional de Electricidad y Energías Limpias, Cuernavaca, Mexico; 3 School of Information Technology, Monash University, Melbourne, Australia; Universita degli Studi del Molise, ITALY

## Abstract

Nowadays, the global energy system is in a transition phase, in which the integration of renewable energy is among the main requirements for attenuating climate change. Wind power is a major alternative to supply clean energy; hence, its widespread penetration is being pursued in all end-use sectors. In particular, it is currently noteworthy to analyze the feasibility of deploying small-scale wind power technology to provide cleaner and cheaper energy in the residential sector. As a first step, a technical assessment must be carried out to provide crucial information to intensive energy consumers, providers of small-scale wind power technology, electric energy distribution utilities, and any other party, to help them decide whether or not to deploy small-scale wind turbines. With this aim, we propose to perform such an analysis using a suitable probabilistic paradigm to solve complex decision-making problems with uncertainty, namely Bayesian Intelligence, since wind resources and energy demands are intermittent variables, properly characterized by probability distribution functions. Then, the problem of determining the technical feasibility can be formulated as an investigation into whether or not small-scale wind turbine technology can produce enough energy to cover the excess demand of intensive energy residential consumers to get off high-priced tariffs. For this purpose, we introduce a novel model based on probabilistic reasoning to assess the suitability of small-scale wind turbine technology to produce the said energy, taking into consideration the availability of wind resources and the energy pricing structure. To demonstrate the usefulness and performance of the proposed model, we consider a case study of deploying 5 and 10 kW wind turbines and analyze the feasibility of their implementation in Mexico, where the energy pricing structure and scattered wind resource availability pose difficult challenges.

## Introduction

### Motivation

The global energy system is undergoing a transition in which the integration of renewable energy is among the key actions being proposed for climate change mitigation [1]. Indeed, the nations participating in the Paris Agreement established reduction goals in green-house emissions in defined dates [2] that have become international law [3]. One way to achieve this is through the intensive use of renewable energy sources. Wind power is currently the second major alternative, behind hydropower, for supplying clean energy. In 2018, it accounted for 24% of the electricity generated by renewables [4].

In the last years, grid-connected Small-Scale Wind Turbine (SSWT) technology, with a generating capacity ranging up to 100 kW, has been used in businesses, factories, farms, and homes as distributed generation to lower carbon footprints and reduce electricity bills [5]. Under suitable wind conditions, SSWTs can generate enough energy to pay back, in periods from a few months to a few years, the carbon emitted during their production, their installation, and operation, and subsequently to compensate for emissions from fossil-fuel generation [6, 7]. In addition, the energy produced by SSWTs can be estimated from a wind speed distribution and the SSWT power curve, provided by the turbine manufacturer. This allows us to evaluate the potential of SSWT technology, in specific sites, to reduce electricity costs by supplying part, the majority, or even exceeding the energy consumption of interested residential consumers [8].

At the same time, consumption is constantly increasing and it is mandatory to look for alternatives to generate energy either from ecological, economic, or practical point of view. In particular, energy consumption is increasing in the residential sector in many countries [9, 10]. This is either due to an increase in the population or due to the use of technology to ensure a level of comfort in everyday life, such as the usage of air conditioning. Moreover, electricity tariffs change depending on many factors such as consumed energy, schedule, month of the year, energy source, and temperature of the site, and they are usually higher for users consuming more energy. In some countries, such as Mexico, the government subsidizes electricity tariffs below specified consumed energy levels, but, if the consumed energy exceeds that level, the electricity price can be much bigger than that subsidized price [11]. Hence, it is important to look for alternatives to provide the required energy. In particular, SSWTs have been considered to generate energy for the residential sector in some countries such as Australia and Chile [12, 13].

So far, we believe that the deployment of SSWT technology by high energy residential consumers can make a contribution to climate change mitigation with the added benefit of reducing electricity bills. Hence, before making any investment, the technical feasibility of SSWT technology to provide enough energy to avoid high electricity tariffs must be demonstrated not with a single crisp number, but with a probability of success due to the many uncertain factors involved in this process. With that in mind, in this paper, we explore the possibility of carrying out a probabilistic assessment on the deployment of SSWT by high energy residential consumers to satisfy their electricity needs, while reducing their electricity bills and making an impact on climate change mitigation.

However, the profitability and convenience of the deployment of small-scale wind power technologies is a complex task from technical, economic, and environmental point of view, among others. In order to make decisions whether or not is feasible to deploy this technology, many aspects that we are not certain about should be taken into account.

Bayesian Networks (BNs) are probabilistic graphical models that allow probabilistic reasoning to deal with problems involving uncertainty [14]. In particular, BNs are one of the tools of Artificial Intelligence (AI) most suitable in simplifying conditionalization, planning decisions under uncertainty and explaining the outcome of stochastic processes. BN technology provides the technical foundation for a truly Bayesian AI. In particular, BNs have been used to solve many open issues in renewable energy systems [15].

Specifically, the Bayesian Decision Networks (BDNs) paradigm extends BNs with actions and utilities (costs and benefits), providing scenario assessment and supporting decision making under uncertainty. In this study, we use this technology to propose a model to assess the technical feasibility of the deployment of SSWT technology to provide energy to high consumption users in the residential sector. Then, we apply this methodology to a case study in Mexico.

Nowadays, Mexico imposes over 22 types of electricity tariffs all over the country. The tariffs are classified according to variables such as the level of voltage and demand of energy, the hourly profile of energy consumption, and the average temperature at the site [16]. The government subsidizes the kWh price up to a certain limit. However, if users exceed the energy limit, they become high residential users (DAC users by its acronym in Spanish) and the kWh price increases up to four times [17]. Therefore, it is important to provide an assessment to decide whether or not it is technically feasible to use SSWTs to help high residential consumption users to reduce the cost of their electricity bills.

The paper is organized as follows. Section 1 mainly introduces the problem and the motivation for addressing it, as well as the objectives that are to be satisfied. Moreover, the section provides relevant antecedents and describes major approaches that have been used to solve similar problems. The novelty of the proposed approach is stated and the structure of the paper is provided to ease understanding. The Intelligent Bayesian Decision-Making section presents key basic concepts of Bayesian artificial intelligence for decision-making. BDNs are introduced as the paradigm for building probabilistic graphical models that will be the core of the decision-making system to assess the deployment of wind turbines in the following sections. The Wind Power Statistics summarizes the essential statistics used to characterize SSWT technology as required to build a decision-making system. Wind resource availability at a given site is characterized by means of histograms of wind velocity measurements and their approximation through Weibull probability distribution functions. At the same time, production of electric power for a given SSWT technology is quantified by specific power curves in terms of wind velocity and the operation stages of the wind turbine. The section Residential Sector in Mexico describes the electricity pricing scheme for the residential sector in Mexico, which mainly embraces household consumers. The kWh price for these consumers depends on the site's tariff class and can vary largely depending on the monthly energy consumption. There are high consumption thresholds (DAC thresholds) defined for each tariff class; below them, kWh price is subsidized, but above them kWh price can be up to four times larger. This circumstance—which is keenly relevant to defining the problem and stablishing the objectives—motivates the study reported in this paper to answer the question: “Is it possible for high-energy consumption (DAC) consumers to stay below DAC thresholds (in a sustainable way) using SSWT?” The section Bayesian Assessment of Small-Scale Wind Power for Reduction of Electric Bills describes the construction of the BDN that is the core of the intelligent decision-making system to assess the deployment of SSWT to avoid DAC tariffs. The BDN is a probabilistic graphical model for reasoning and making decisions under uncertainty that requires representing knowledge in the form of probability distribution functions. Therefore, detailed procedures are given to construct discrete probability distributions of the energy produced by SSWT, the discrete power distribution of SSWT technology, the monthly probability distribution of energy produced in a given site, and the probability distribution of the average consumed energy by the consumer. Additionally, a probabilistic measure of suitability is defined for the decision-making system to have a criterion to decide with a degree of probability whether or not the usage of SSWT is appropriate to avoid DAC tariffs. The Case Studies in Mexico section provides case studies on the assessment of deploying 5- and 10-kW SSWTs for DAC consumers to avoid high energy consumption tariffs in various cities of interest in Mexico, to demonstrate the feasibility of the proposed intelligent decision-making system. The BDN is realized by programming it in the software Netica using real wind and electricity data. Results are presented and compared for all cities being considered. Also, a comparison with other published related work using different methodologies is performed. Finally, the last section presents the conclusions of this study.

### Related work

Evaluations of carrying out the installation of wind turbines have been done under multiple approaches. A precise assessment of wind resources is very important for the installation of wind energy technologies since a minor deviation in wind speed causes a large deviation in the output power of wind turbines [18–20]. Indeed, Rodríguez *et al*. [21] show that wind speed percentage errors of 10% imply an error of 5% in the power output estimates. Wind resource assessment has been made in many countries. For instance, wind-speed dynamics have been incorporated for project evaluation in the USA [22]. Likewise, wind resource assessment for Australia has been studied by Hallgren *et al*. [23] and along the Western Coast of Thailand by Niyomtham *et al*. [24]. Additionally, the assessment for wind and solar power in the European Union has been considered by Buttler *et al*. [25].

Similarly, decision-making models have been developed for locating wind turbines. This is the case in the study by Gagliano *et al*. [26] where a methodology to exploit the fields of wind flow using dynamic computational fluids—considering the Geographic Information System (GIS)—in urban environments is developed and presented in a pilot study for the capital of Sicily. Lee *et al*. [27] presented a multi-criteria decision-making system, based on a hierarchical analytical process with benefits, opportunities, costs, and risks, in order to select suitable wind farm projects and apply the model to a case study in China. In a study by Tegou *et al*. [28], a framework for assessing the suitability of land for the establishment of wind farms, combining multi-criteria decision making by means of a GIS analysis, is performed. A hierarchical method with analytical and diffuse processes is presented by Goh *et al*. [29], in order to evaluate priority criteria of site selection for deploying wind farms, and the method is applied to a case study in Malaysia. Similarly, Sánchez-Lozano *et al*. [30] created a diffuse method to make multi-criteria decisions for offshore wind farm site selection and evaluation in the southeast of Spain. Another multi-criteria analysis method for site selection of wind farms using GIS is presented by Haaren and Fthenakis [31], with a case study in the state of New York. Finally, the same method is applied to analyze the suitability of wind farm locations in Ecuador [32].

Another approach to evaluate the installation of wind turbines is through techno-economic feasibility assessments. For example, in a study using data from an atmospheric monitoring program in Mexico City, Rodriguez-Hernandez *et al*. [33] evaluate 28 models of small wind turbines at 18 sites in the city in 2019. Residential users were considered as potential users, but it was found out that only two sites had positive economic evaluations independently of the achieved reductions in CO_2_ emissions. A similar study was performed on the campus of a university in Switzerland [34], where the potential of wind power production was statistically analyzed by using average wind speed into the four seasons of the year. In 2008, a report on the political vision and practice guidelines for SSWT technology were prepared in the UK by Carbon Trust [35]. The report considers carbon savings, the potential of wind turbines at different UK sites, as well as local policies for sites under consideration. In 2011, Reuther *et al*. [36] carried out a feasibility study of SSWTs for residential users in New Zealand. Residential electricity demand, policies, and regulations, as well as the on-site wind resource, are considered. An analysis of the payback period for small wind turbines in Istanbul is presented by Ugur *et al*. [37]. Performance and feasibility studies of small wind turbines for the European Union were carried out in 2014 [38]. The NREL in 2015 [39] published a guide for site evaluation of small wind turbines considering aspects such as wind resources, topography, land use, security, environmental conditions and policies of utilities for the USA. Likewise, an economic feasibility analysis of small wind turbines for residential consumers in Egypt is performed by Abdelhady *et al*. [40].

## Intelligent Bayesian decision-making

### Decision networks

BNs support reasoning under uncertainty, by computing posterior probability distributions for sets of query nodes, given values for some evidence nodes. BNs can be extended to BDNs to support decision-making. BDNs, also called influence diagrams, combine the probabilistic reasoning of BNs, with capabilities to make decisions that maximize the expected utility. A decision network consists of three types of nodes: a) chance nodes symbolized by an oval shape representing random variables; b) decision nodes symbolized by a rectangular shape representing the actions that the decision-maker must choose between; and c) utility nodes symbolized by a diamond shape representing the utility functions measuring the preferences over a set of decisions [41].

**Fig 1:**
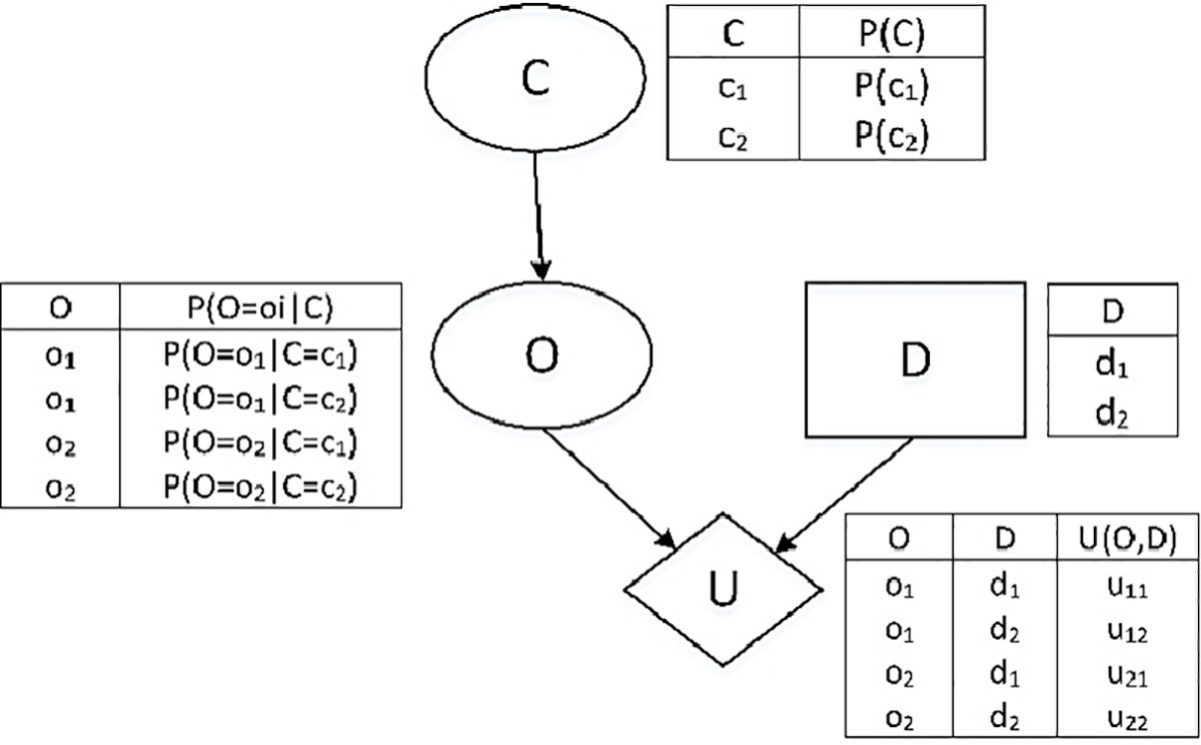
A BDN and its conditional tables.

For instance, consider a generic BDN as shown in Fig 1. Nodes *C* and *O* correspond to chance nodes, *D* corresponds to a decision node, and *U* to utility node. The variable corresponding to the *C* node can be in two states corresponding to *c*_1_ and *c*_2_, with a priori probabilities, *P*(*c*_1_) and *P*(*c*_2_), respectively, shown in the table next to the node.

The *O* node corresponds to an action with two possible states, *o*_1_ and *o*_2_, that depends on *C*. A Conditional Probability Table (CPT) is associated with this node, and corresponds to the probability distributions over the states of *O* given the states of *C*, given by *P*(*O* = *o*_*i*_|*C*) for *i* = 1,2, and shown in the table at the left-hand side of the node. Then, the probability distributions of the states of *O* are given by

$$P(O=o_{i})=\sum_{j}P(C=c_{j})\times P(O=o_{i}|C=c_{j}),$$ (1)

In general, a BN computes the probabilities attached to a node state given the state of one or several variables, becoming a powerful modeling tool for complex systems [34]. Hence, for a BN containing *n* nodes, *X*_1_ to *X*_*n*_, a particular value of the joint distribution of each variable at a specific state *P*(*X*_1_ = *x*_1_,*X*_2_ = *x*_2_,…,*X*_*n*_ = *x*_*n*_)≡*P*(*x*_1_,*x*_2_,…,*x*_*n*_) is given by

$$P(x_{1},x_{2},\ldots ,x_{n})=\prod_{i}P(x_{i}|Parents(X_{i})),$$ (2)

where *P*(*x*_*i*_|*Parents*(*X*_*i*_)) is the conditional probability that *X*_*i*_ = *x*_*i*_ given the states of the node parents, *Parents*(*X*_*i*_).

The *D* node corresponds to a binary decision node with two values *d*_1_ and *d*_2_, as represented in the table to the right of the node.

Finally, the *U* node corresponds to the utility node with an associated utility table for the different states of *O* and *D*, shown at the right of the node. The utility values of this node, *u*_*i*,*j*_, are calculated from the utility function of the problem in question.

The expected utility *EU* for a given value of the decision node, for example, *D* = *d*_*i*_ is calculated as

$$EU(D)=\sum_{j}P(O)\times U(O|D),$$ (3)

where the sum over the *j* index refers to the sum over all the states of the action corresponding to node *O*, namely, *o*_1_,*o*_2_,…,*o*_*j*_. *U*(*O*|*D*) is the utility function and provides the utility of each of the outcome states, given that the decision *D* is taken. It can be given by values defined in an associated utility table which represents the preferences of every state, as shown in Fig 1, or by a function whose variables are the states of *O* and *D* and it depends on the nature of the problem on consideration. For example, the expected utilities for the case that the decision is *D* = *d*_1_ or *D* = *d*_2_ are calculated as follows

$$EU(d_{1})=P(o_{1})\times U(o_{1}|d_{1})+P(o_{2})\times U(o_{2}|d_{1}),$$ (4)

$$EU(d_{2})=P(o_{1})\times U(o_{1}|d_{2})+P(o_{2})\times U(o_{2}|d_{2}),$$ (5)

Then, a BDN calculates the conditional probabilities and expected utilities for the different scenarios and returns the action (decision) with the highest expected utility. The construction of a BDN involves the modeling of as many aspects as possible of the real world involving the definition of the variables and their values or states, the graph structure, the parameters, the available actions and decisions, and their impact, and the utility nodes and their dependencies, among others.

### Knowledge Engineering with BNs

Knowledge Engineering consists of integrating expert knowledge into computer systems to solve problems requiring a high level of human expertise. The goal is to construct the so-called expert system such that it reasons, learns, and solves complex problems in a way similar to that of a human expert. In this work, the result of the Knowledge Engineering process is an expert system that provides an assessment of the technical feasibility of SSWT technology to avoid high electricity bills for high residential consumption users. As it is shown in the following sections, this process involves constructing a model that is sufficiently complex to realistically represent the problem features, while being simple enough to be represented by a probabilistic graphical model.

The Knowledge Engineering with Bayesian Networks (KEBN) lifecycle model is a suitable way to construct the expert system and involves the following steps, as described in Chapter 10 of the book by Korb and Nicholson [42]: a) construction of the BDN which consists of defining the variables, the network structure, and its parameters from expert knowledge or available data; b) validation of the network in order to evaluate it through a sensitivity analysis and accuracy tests; c) field-testing to check the actual use of the network either by in-house staff or by a friendly-user; d) industrial use to implement the network in real practice; and e) refinement of the network to improve it or fix bugs.

In this work, we deal with the first step of the KEBN lifecycle. Within steps a) and b), there is a further iterative and incremental modeling process that we have followed in our modeling. Our aim is to go through the entire cycle but we want to involve more aspects relevant to the problem in future work. In the next section, we describe the problem to be modeled, namely the high tariffs in electricity bills for residential consumption users. Then, the Residential Sector in Mexico section describes the construction of the expert system that provides an assessment of the technical feasibility of SSW technology to get rid of high electricity bills in the residential electricity sector.

## Wind power statistics

### Wind resources

**Fig 2:**
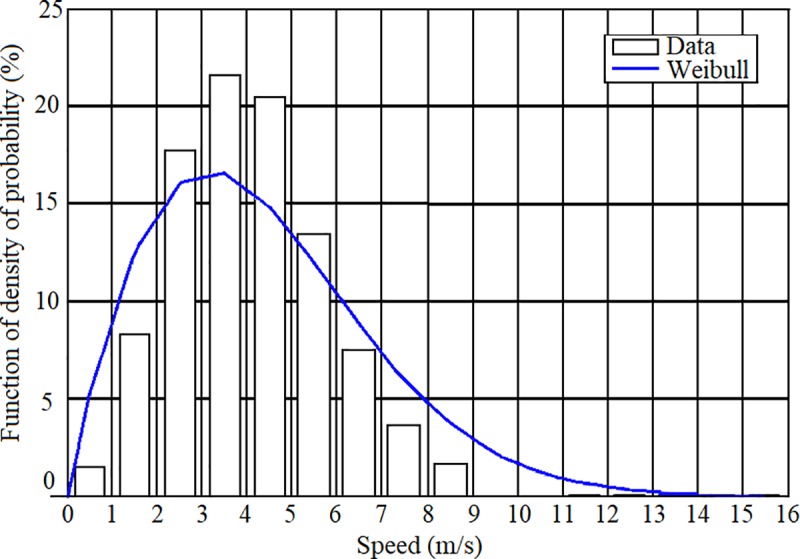
Speed histogram and Weibull probability distribution for wind resources.

A wind turbine generates wind power by converting wind kinetic energy into electric energy. The kinetic energy of wind depends on the wind velocity. However, since wind velocity is not constant, in order to calculate the mean power delivered by a wind turbine it is necessary to know the probability density distribution of wind speed. Every site exhibits different wind speeds throughout the day and throughout the year. Therefore, wind resources at a site can be characterized by a wind speed frequency histogram corresponding to the counts of measurements corresponding to different wind speeds at the site through the year. Then, the histogram can be fitted to a probability density distribution of the wind speed. At most sites, the histogram is well fitted to a Weibull probability distribution, shown in Fig 2, and given by Papoulis and Unnikrishna [43],

$$f(v)=\left\{ \begin{array}{r} \frac{k}{\lambda }{\left(\frac{v}{\lambda }\right)}^{k-1}e^{{-(v/\lambda )}^{k}},v\geq 0\\ 0,v<0 \end{array},\right.$$ (6)

where *v* corresponds to the wind speed, and *k*>0 and *λ*≥0 are the shape and scale parameters, respectively, that characterize the probability distribution.

### Wind power

**Fig 3:**
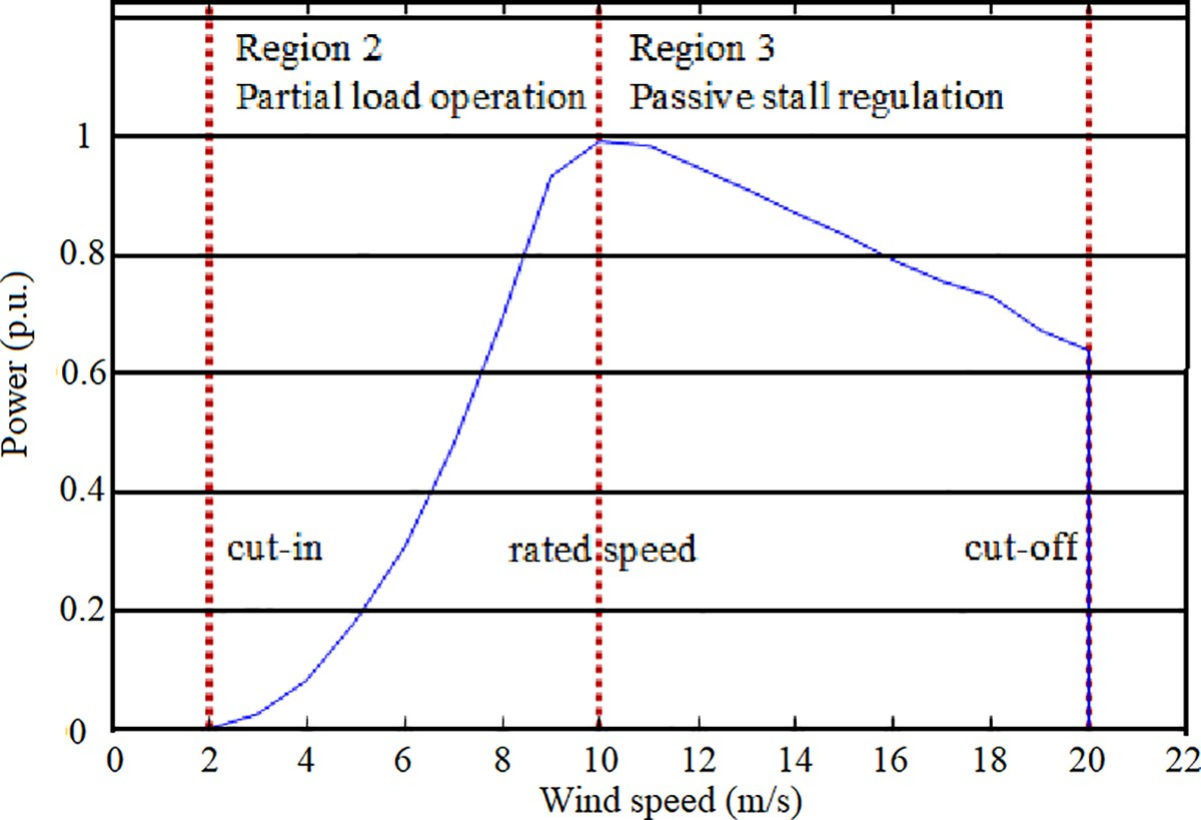
Power curve *P*(*v*) of an SSWT generator.

Electric power generated by a wind turbine is given by its power curve and depends on the wind speed, as demonstrated by Walker and Swift [44]. Fig 3 shows a typical power curve corresponding to the generated power by a wind turbine as a function of the wind speed. There are two operation regimes: a) power is generated at a cut-in wind speed which typically ranks between 3 and 4 m/s; then, the wind turbine operates at partial load between the cut-in speed and the rated speed; and b) full load operation starts at the rated speed and stops at the cut-off speed due to security reasons. In this region, the wind turbine generates its nominal power.

The mean power generated by the wind turbine from its power curve is given by

$$P=\int_{v_{i}}^{v_{f}}P(v)\;f(v)\;dv,$$ (7)

where *P* is the mean generated power, *P*(*v*) is the power curve as a function of the wind speed *v*, *f*(*v*) is the Weibull probability density distribution of the wind speed and, *v*_*i*_ and *v*_*f*_ are the initial and final operation wind speeds.

Wind turbines can produce power from a few kW up to 10 MW and they are classified in four classes depending on their operation speed according to the International Standard IEC 61400 [45]. Table 1 shows the wind turbine class and the corresponding average operation wind speed.

**Table 1:** Wind turbine classes.

Wind turbine class	Annual average wind speed (m/s)
**I (High wind)**	10
**II (Medium wind)**	8.5
**III (Low wind)**	7.8
**IV (Very low wind)**	6

In recent years, SSWTs have attracted attention because of their potential to provide clean energy for microgeneration. In particular, SSWT technology could be an ad-hoc solution to generate power for communities with good wind resources.

## Residential sector in Mexico

The Federal Electricity Commission (Comisión Federal de Electricidad, CFE) is the state-owned electric utility of Mexico and it is the country´s dominant electric company. CFE was founded in 1937 by the Federal Government and it is responsible for controlling, generating, transmitting and commercializing electric power throughout the country [46]. CFE costumers cover the residential, business and industry sectors: the residential sector mainly covers households; the business sector is made up of companies; and the industry sector refers to industries. Every sector has its own tariff scheme for the price of electrical power (in kWh). The residential sector is one of the main energy consumers among all consuming energy sectors. In the last twenty years, its share of total energy usage was between 20 and 15%. Energy consumed in this sector is used for household welfare providing services arising from necessity up to luxury consumption, such as cooking, heating, lighting, cooling and entertainment. Electricity, LPG, natural gas, kerosene and firewood are the main energy sources to satisfy the residential energy demand. In particular, electricity underwent the biggest growth rate over the last two decades, and thus its price increased. As it was mentioned above, Mexico is going over an energy transition period. Reduction of the price of electricity and increasing the participation of clean energies in the generation of electricity are among the main goals of the Mexican Energy Ministry for the next years. The price of electricity in the residential sector depends on the consumption and the summer's minimal average temperature at the site. Table 2 shows the average summer temperature corresponding to each electricity rate or type of user as well as the maximum energy allowed.

**Table 2:** Residential electricity tariffs in Mexico.

Tariff	Average Temperature (°C)	DAC threshold (kWh/month)
**1A**	25	300
**1B**	28	400
**1C**	30	850
**1D**	31	1000
**1E**	32	2000
**1F**	33	2500

Once the energy limit (DAC threshold) has been exceeded in each user type, the user becomes a DAC (high residential consumer by its Spanish acronym). Consumption below the DAC threshold is subsidized, but once a user reaches a DAC tariff, the electricity price stops being subsidized and can increase by up to four times. However, high consumption users could avoid DAC tariffs using micro renewable power in order not to exceed the energy consumption limit but still meeting their consumption needs.

The tariff system has changed in the last years. In 2016 there was a kWh price for each tariff. In the following years, the tariff scheme changed and the electricity price now depends on several factors: a) the tariff, b) the beginning of the summer, c) the month under consideration and, d) the amount of energy consumed: basic, intermediate or surplus. Furthermore, in addition to the fact that the tariff system has become more complex, the price of electricity, and in particular the DAC tariff, continues to increase over the years. Therefore, it becomes mandatory to look for alternatives to provide energy in order to satisfy electricity demand. In the next section, we present a decision-making model to assess the use of SSWTs to meet the high-energy demand in sites with wind potential.

## Bayesian assessment of small-scale wind power for the reduction of electric bills

In this section, we construct a BDN model (system) to assess the deployment of SSWTs to reduce electric bills for high residential consumers. In the following subsections, we describe the modeling of small-scale wind energy production (§5.1) and residential consumption (§5.2) as probabilistic distributions. We use these probabilities (§5.1) to define a measure of “*suitability*” to provide an assessment of the technical feasibility of the exploitation of SSWT technology for a given site. Then, we show how these distributions can be represented in the BDN to assess decision-making for the exploitation of wind energy for residential use, explicitly showing the economic impact of the decisions.

### Modeling small-scale wind energy production

The first step in this work is to construct the probability distribution of the energy produced by SSWTs in order to implement it in the BDN. The initial data to start with are the following:

- The Weibull parameters, *k*, and *λ*, for the site of interest are provided by the site prospector.
- The power curve, *P*(*v*), corresponding to the wind turbine under consideration is given by the manufacturer.

At the same time, the following assumptions are made in order to obtain the probability distribution of the energy produced by SSWTs:

1. The Weibull parameters were obtained from samples taken during an entire year, so that the percentage of wind speed measurements corresponds to the same percentage of wind power.
2. The SSWT was available during the entire year, i.e., we consider a capacity factor equal to one. This corresponds to considering that all wind power available is converted to electric power.
3. In a first approximation, we consider that the wind behavior is statistically similar every month, such that the monthly wind speed distribution is similar to the annual one.

**Fig 4:**
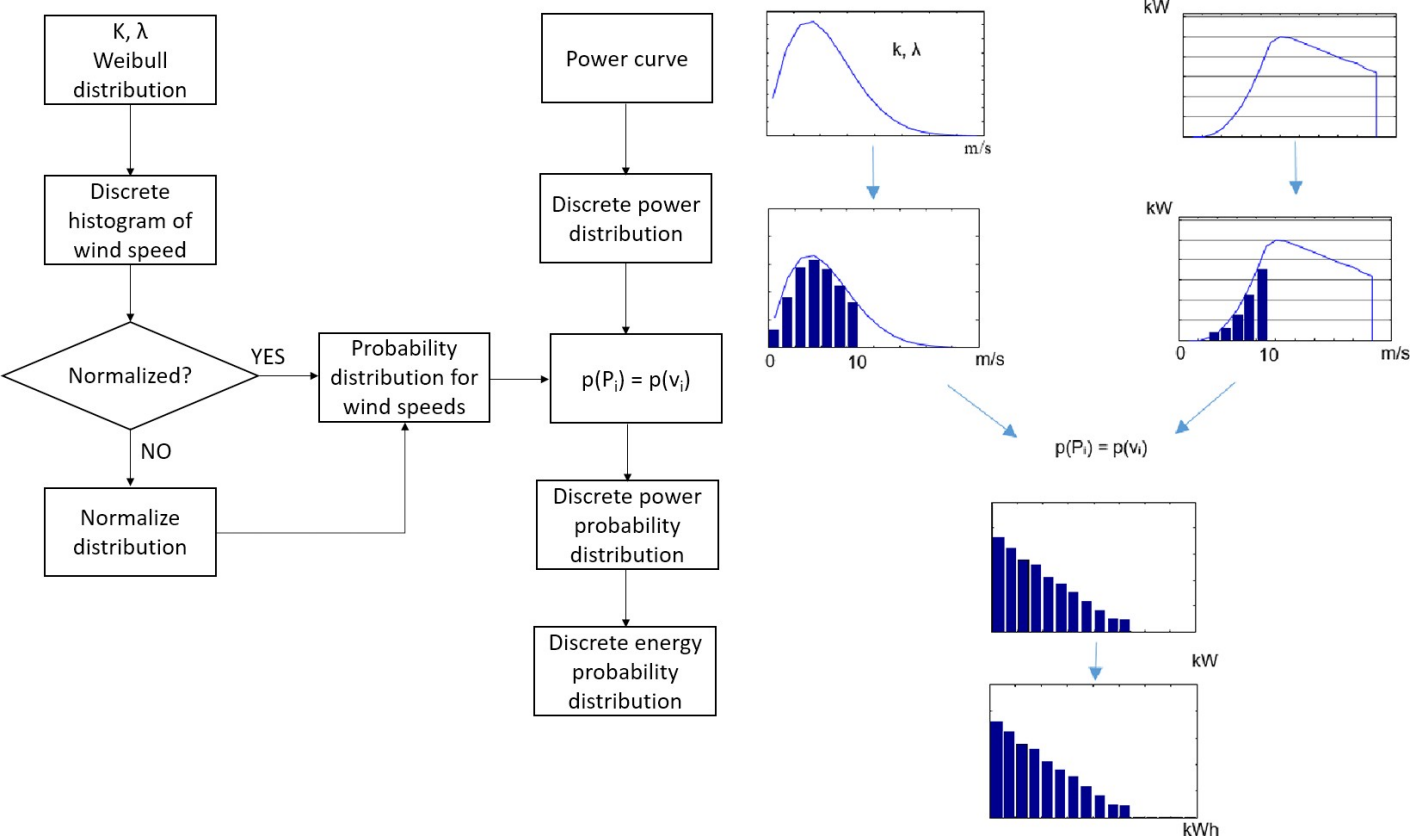
Methodology for modeling the probability distribution of energy production with the wind turbine generator: (a) flow chart of the procedure and (b) schematic description of the procedure.

The methodology to model the wind power generation is a two-tier procedure, as shown in Fig 4. Fig 4(A) shows the flow chart of the procedure and Fig 4(B) describes it schematically. First, the Weibull distribution function of wind speed, *f*(*v*), at the site is obtained using the given Weibull parameters *k* and *λ*. The next step consists of fitting a histogram of wind speeds to the distribution function. This operation is equivalent to discretizing *f*(*v*) into *i*_*max*_ bins to obtain the discrete distribution *f*(*v*_*i*_), where *i* = 1,2,…,*i*_*max*_. Notice that this process corresponds to the inverse practice usually done to determine Weibull parameters from a set of annual data of wind speeds at the site [47]. Usually, the obtained wind speed discrete distribution is normalized, but in case it is not, it must be normalized in order to get the discrete probability distribution for wind speeds, *p*(*v*_*i*_) such that

$$\sum_{i=1}^{i_{max}}p(v_{i})=1.$$ (8)

It should be noted that for small-scale wind power applications only the range of wind speeds of 0–10 m/s is of interest. Hence, the bins are defined as consecutive, adjacent, non-overlapping intervals and often (but not necessarily) of equal size in the range of 0–10 m/s. The former procedure corresponds to the left-hand side of the flow chart and scheme in Fig 4.

On the other side, the given power curve function, *P*(*v*), must be discretized using the same bins as for the discrete wind speed distribution, *f*(*v*_*i*_), to obtain the discrete power distribution *P*(*v*_*i*_). It is worth noting that in the wind speed range of interest, i.e., 0–10 m/s, the power curve has not reached yet its maximum, as shown in Fig 4, the produced power monotonically increases with wind speed, and the turbine blades are kept at the zero position by the control system to capture the most energy from the wind.

Next, the discrete probability distribution for wind speeds, *p*(*v*_*i*_), and the discrete power curve, *P*(*v*_*i*_), are used to construct the monthly discrete probability power distribution, *p*(*P*_*i*_), at the site of interest for a given small-scale wind technology, as follows. From the discrete power curve, *P*(*v*_*i*_), it is known that the wind turbine generator produces power *P*_*i*_ at wind speed *v*_*i*_. Then, considering assumption 2, the probability to produce power *P*_*i*_,*p*(*P*_*i*_), is equal to the probability of the corresponding wind speed *p*(*v*_*i*_),

$$p(P_{i})=p(v_{i}),$$ (9)

for *i* = 1,2,…,*i*_*max*_. Note that from Eq (9) it follows that the discrete probability power distribution satisfies

$$\sum_{i=1}^{i_{max}}p(P_{i})=1.$$ (10)

Finally, the assumption 3 is used in order to obtain the monthly discrete probability energy distribution, $p({E_{W}}_{i})$, of produced energy by the wind turbine generator in kWh per month. First, we calculate the monthly energy, ${E_{W}}_{i}$, corresponding to each power bin, *P*_*i*_, as follows

$${E_{W}}_{i}=P_{i}\times 30\times 24,$$ (11)

where we have assumed that all months are made of 30 days. Then, in order to construct $p({E_{W}}_{i})$, each energy bin is located at ${E_{W}}_{i}$ and its probability, $p({E_{W}}_{i})$, is equal to the probability of the corresponding power *p*(*P*_*i*_),

$$p({E_{W}}_{i})=p(P_{i}).$$ (12)

Then, the looked-for discrete probability energy distribution also satisfies

$$\sum_{i=1}^{i_{max}}p({E_{W}}_{i})=1.$$ (13)

Hence, the expected energy, *E*_*W*_, produced per month by the wind turbine generator is given by

$$E_{W}=\sum_{i=1}^{i_{max}}{E_{W}}_{i}\;p({E_{W}}_{i}).$$ (14)

This procedure establishes a methodology to construct the monthly probability distribution of energy produced on a site, given the Weibull parameters of the wind resource and the wind turbine power curve. In the next subsection, we describe the treatment of the consumed energy in the residential sector for the purposes of the model. It is important to highlight that this formalism is required, and facilitates building a BDN to model the system.

### Modeling the energy consumption in the residential sector

In this section, we calculate the probability distribution of the average consumed energy, *E*_*C*_, by the user. This probability distribution is necessary in order to be implemented in the BDN, constructed in the next subsection, that models the system to assess the use of SSWT technology in order to avoid high electricity costs.

The initial data to start with are the time series of monthly data of average consumed energy in one year, per high residential consumer, at a given site, *E*_*C*_(*t*), where *t* = 1,2,…,12 stands for the months of the year.

In addition, the following assumptions are made:

1. The consumed energy per high residential consumer will grow, or at least remain the same, in the coming years.
2. Consumers retain their tariffs for the entire year. Therefore, we consider DAC users for the analysis.

**Fig 5:**
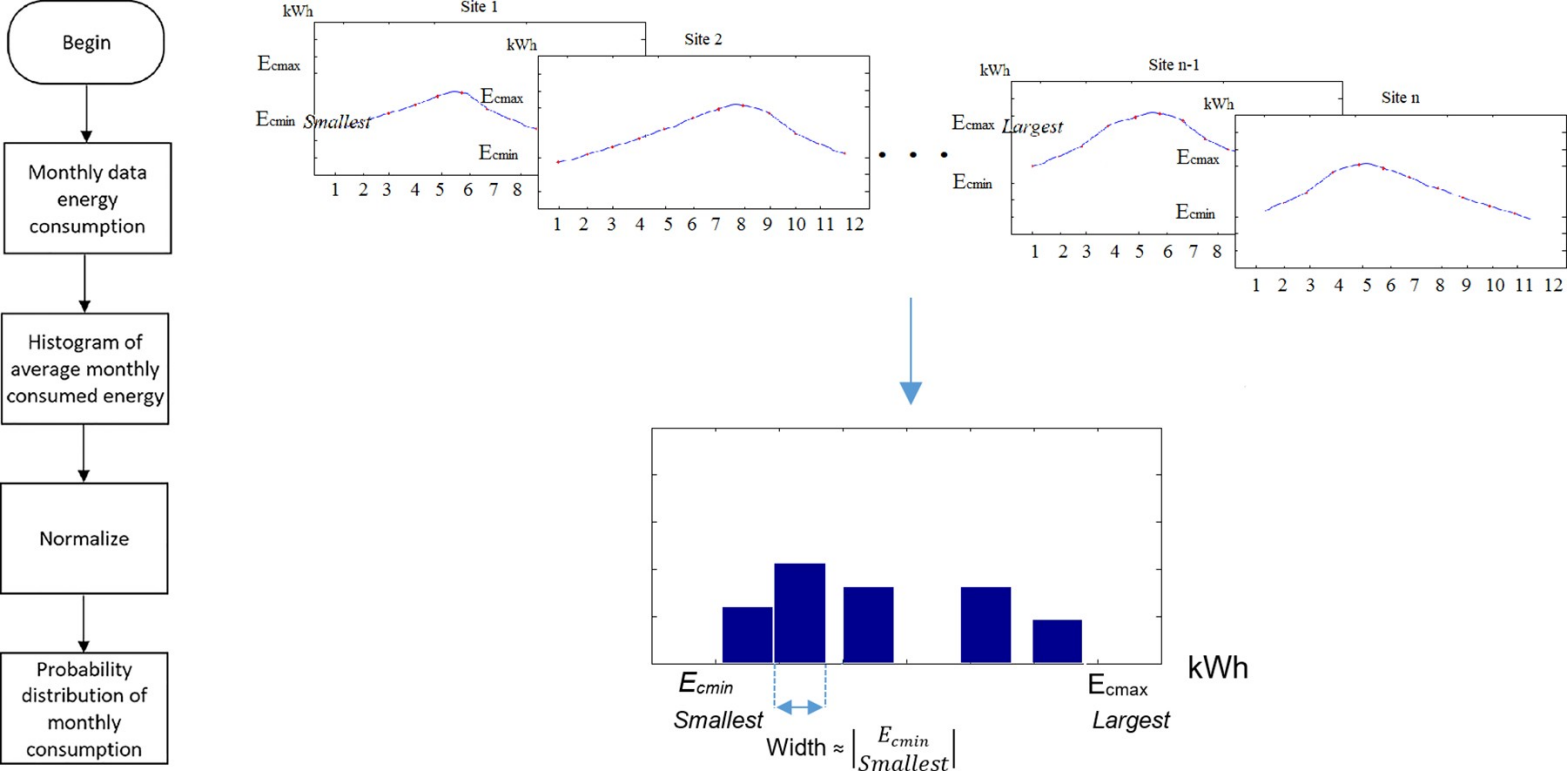
Methodology for the modeling of the probability distribution for the monthly energy consumption: (a) flow chart of the procedure and (b) schematic approach of the procedure.

Fig 5 shows the methodology for obtaining the probability distribution of the average monthly consumed energy *p*(*E*_*C*_) per DAC user. Fig 5(A) shows the flow chart of the procedure and Fig 5(B) describes it schematically. From the time series of monthly data of consumed energy, *E*_*C*_(*t*), we can construct a histogram in the following way:

- Define the range of the histogram that includes the probability distribution functions of the average monthly consumed energy of all sites. In order to do that we must keep in mind that every site has its own minimum and maximum monthly average energy consumption, $E_{C_{min}}$, and $E_{C_{max}}$. Then, to define the range of the histogram, we consider the smallest $E_{C_{min}}$ and the largest $E_{C_{max}}$ of all sites. Then, the range lower limit is placed on 0 and the range upper limit is located several kWh above the largest $E_{C_{max}}$, so that meaningful bins can be defined in the next step.
- Define the width of the bins. Given the range of the histogram and the smallest $E_{C_{min}}$, one proposes a width length close to the magnitude of the smallest $E_{C_{min}}$ so that: i) the smallest data of energy consumption shows up in the histograms, and ii) all histograms get allocated to several bins. Note that bins are specified as consecutive, non-overlapping, adjacent and often (but not required to be) of equal size intervals.
- Determine the occurrence frequencies of consumed energy. From the time series, *E*_*C*_(*t*) for *t* = 1,2,…,12, count the number of times each element of the series falls into the bins. Thus, by depicting the frequencies of observations the histogram of the consumed energy is constructed.

Note that a few iterations of this process may be required. Finally, the probability distribution for the monthly average consumed energy, *p*(*E*_*C*_), is found by normalizing the histogram such that the sum of heights equals 1. Then, we obtain the probability distribution satisfying,

$$\sum_{j=1}^{j_{max}}p(E_{Cj})=1,$$ (15)

where *j*_*max*_ is the number of bins.

### Condition for small-scale wind power to reduce electricity bills

In order to decide whether the usage of SSWTs is suitable to avoid DAC tariffs, one must determine if the produced energy from the SSWT generator helps the consumer to satisfy their energy demand without exceeding the energy limit *E*_*DAC*_. Therefore, we consider the excess energy *E*_*X*_ defined as

$$E_{X}=E_{C}-E_{DAC}.$$ (16)

Then, we probabilistically compare the excess energy, *E*_*X*_, with the energy produced by the SSWT generator, *E*_*W*_. The probability of *E*_*X*_ being smaller than *E*_*W*_ provides the probability of small-scale wind technology to be appropriate to avoid DAC tariffs. Complementarily, the probability of *E*_*X*_ being larger than *E*_*W*_ provides the probability of small-scale wind technology to be unsuitable to avoid DAC tariffs such that

$$suitability=\left\{ \begin{array}{c} p(yes)=p(E_{x}\leq E_{w})\\ p(no)=p(E_{x}>E_{w}) \end{array}.\right.$$ (17)

In this way, the *suitability* provides a probabilistic measure to assess the technical feasibility of the exploitation of small-scale wind technology for a given site. In the next section, we construct the decision-making system to assess the technical feasibility of the use of SSWT technology to reduce the cost of electricity bills.

### Construction of the decision-making system for Bayesian assessment

In the previous subsections, we modeled the main components for building the BDN to assess the technical feasibility of the exploitation of SSW technology through probabilistic reasoning. In a BN, as described in the Intelligent Bayesian Decision-Making section, variables are represented by nodes and the direct dependency between them is represented by arcs connecting the nodes. Each variable takes on states specified by conditional probability distributions. Below, we describe the construction of the BDN that performs the assessment of the exploitation of the SSW technology to avoid DAC tariffs.

**Fig 6:**
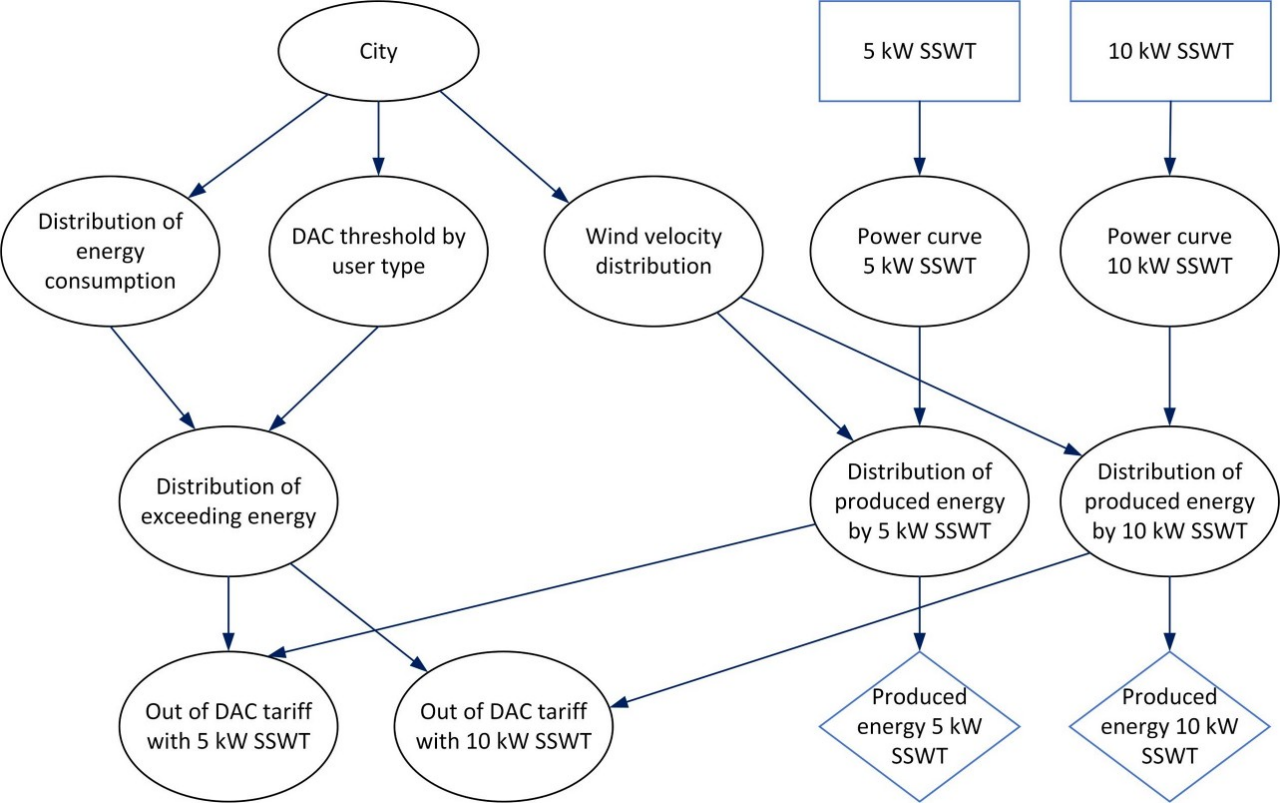
BDN for the assessment of small-scale wind technology deployment.

Fig 6 shows the BDN for the assessment of SSWT technology deployment to avoid DAC tariffs. We start at the top of the BDN. The parent node “City” represents the variable with states corresponding to the cities being considered. This node is used to select the city for which we want to perform the assessment. Then, the available wind resource is introduced in the right branch of the diagram. The chance node “Wind velocity distribution” is directly dependent on the parent “City” as every city is characterized by its own wind speed distribution. Then, for a selected city of the parent node “City”, the probability distribution of the wind speeds, *p*(*v*_*i*_), at this city is assigned. Next, the decision nodes “5 kW SSWT” and “10 kW SSWT”, represented by the blue rectangles, are introduced to select the scenarios of the what-if analysis using 5 or 10 kW SSWTs. The chance nodes “5 kW SSWT Power curve” and “10 kW SSWT Power curve” introduce the discrete power curves, *P*(*v*_*i*_), corresponding to SSWT generators of 5 or 10 kW, respectively. Note that these two decision nodes represent mutually exclusive decisions, so we could instead represent them in a single decision node with three alternatives (no wind power, 5 kW wind, or 10 kW wind power). However, we’ve chosen to use two separate nodes so that the impact on the two power curve chance nodes is clearer. The nodes “Distribution of produced energy 5 kW SSWT” and “Distribution of produced energy 10 kW SSWT” calculate the probability distribution for the produced energy, $p({E_{W}}_{i})$, for each of the turbine generators using Eq (11). At the end of both branches, the utility nodes “Produced energy 5 kW SSWT” and “Produced energy 10 kW SSWT”, depicted by the blue rhombuses, represent the produced energy *E*_*W*_ by each SSWT generator as given by Eq (14).

The left branch of the BDN deals with the consumed energy. In every city, DAC consumers come from different types of users that consume different amounts of energy. Therefore, we introduce the nodes “Distribution of energy consumption” and “DAC threshold by user type” as directly dependent on the selected city. Then, for a selected city, a DAC threshold is selected in the “DAC threshold by user type” node, corresponding to the dominant user type of the city according to Table 2. Likewise, for a given city, a probability distribution of the average monthly consumed energy per DAC user, $p({E_{C}}_{j})$, is selected in the “Distribution of energy consumption” node. Next, the “Distribution of exceeding energy” node calculates the excess energy, *E*_*X*_, as given by Eq (11). The states of this node are the probability distributions *p*(*E*_*X*_) given by the conditional probability *p*(*E*_*X*_|*E*_*C*_,*E*_*DAC*_).

Finally, the nodes “OUT of DAC tariff with 5 kW SSWT” and “OUT of DAC tariff with 10 kW SSWT” calculate the *suitability* defined by Eq (17). The states of these nodes are *yes* or no, which correspond to the conditional probability that *E*_*X*_ is smaller or larger than *E*_*W*_ as defined by Eq (16).

In the next section, we demonstrate and evaluate the BDN methodology by applying it to cities in Mexico which could have adequate conditions to use SSWT technology to avoid DAC tariffs.

## Case studies in Mexico

**Fig 7:**
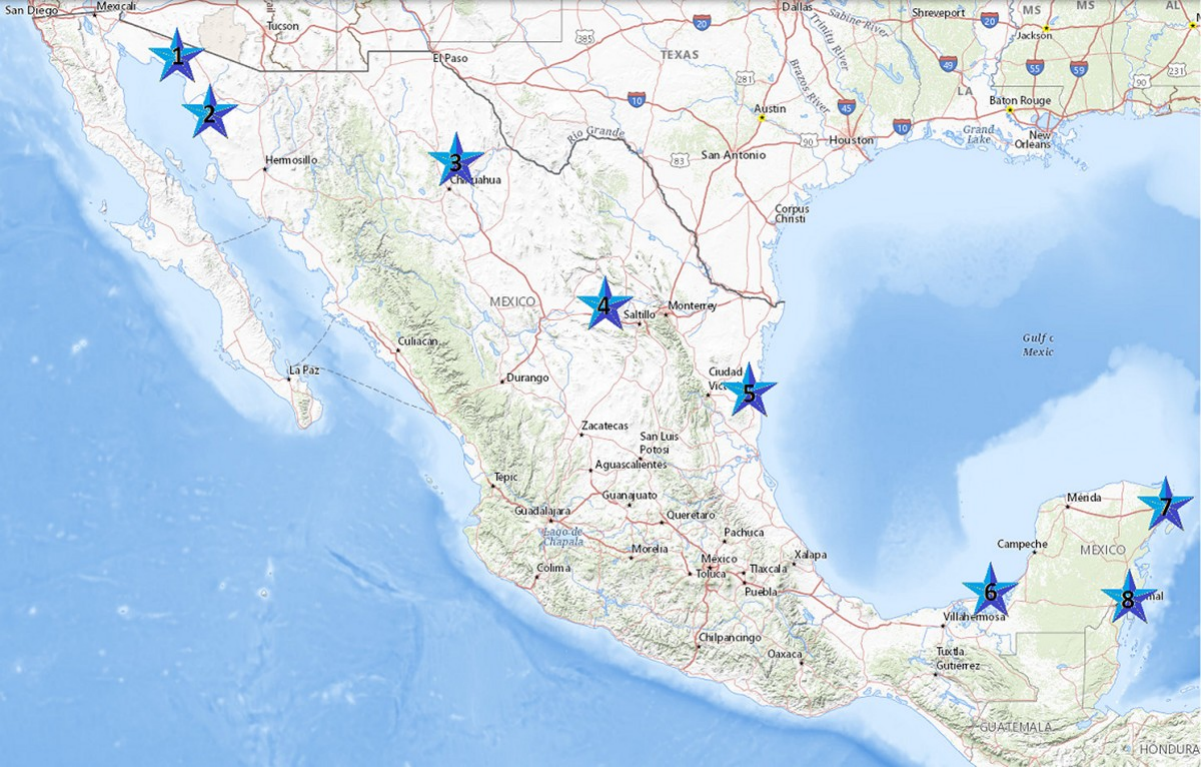
Cities in Mexico with adequate wind conditions to produce SSW energy. Map obtained from USGS National Map Viewer (public domain): http://viewer.nationalmap.gov/viewer/.

In this section, we apply the previous methodology to assess the deployment of SSWTs to reduce electric bills for high residential consumers in cities in Mexico where the wind resource is ideal for SSWT technology and there is high consumption in the residential sector. Fig 7 shows the cities under consideration, starting from the left top side on the map, San Luis Rio, Puerto Peñasco, Chihuahua, Parras, Tampico, Ciudad del Carmen, Cancún, and Cozumel.

### Input data

As mention in subsection Wind Power, there is an electricity tariff, or user type, depending on the maximum average temperature in summer (Table 2). There is more than one user type in most of the places, however, Table 3 shows the dominant user types for the cities under study.

**Table 3:** Electricity user type for the cities shown in Fig 7.

City	User Type
**Puerto Peñasco**	1E
**Parras**	1A
**Tampico**	1C
**Ciudad del Carmen**	1C
**Cancún**	1D
**San Luis Rio**	1F
**Chihuahua**	1B
**Cozumel**	1D

Data obtained from Federal Electricity Commission (CFE), “residential tariff schemes”, available at *https*:*//app*.*cfe*.*mx/Aplicaciones/CCFE/Tarifas/TarifasCRECasa/Casa*.*aspx* and accessed in November 2019.

**Fig 8:**
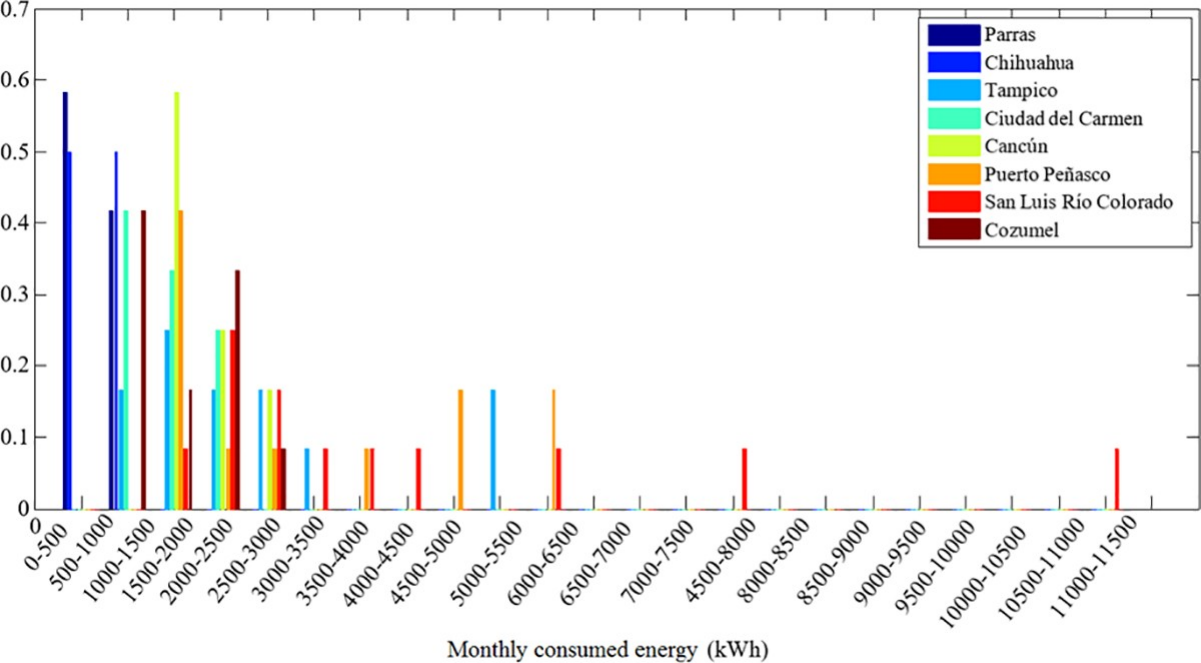
Distribution of the energy consumed per month by DAC users in the cities shown in Fig 6.

Likewise, the discrete probability distribution of the average monthly consumed energy by the DAC users in these cities is shown in Fig 8. These distributions are obtained from the data presented in the Supporting Information.

The wind resource at these cities is characterized by an annual Weibull distribution given by the shape, *k*, and scale, *λ*, parameters shown in Table 4. Using these parameters the Weibull distribution function of wind speed, *f*(*v*), can be plotted to obtain the probability distribution of wind speed at these sites, as shown in the Supporting Information. The average wind speed at these sites is also reported in Table 4.

**Table 4:** Mean wind speed and shape and scale parameters of the Weibull distribution for the cities under study.

City	v[m/s]	*k*	*λ*
**Puerto Peñasco**	4.90	2.181	5.140
**Parras**	4.36	2.215	5.415
**Tampico**	3.94	1.877	4.352
**Ciudad del Carmen**	5.18	2.360	5.164
**Cancún**	4.23	2.382	4.282
**San Luis Rio**	4.66	2.303	4.899
**Chihuahua**	2.78	1.718	3.418
**Cozumel**	4.12	2.298	4.331

These data were obtained from National Institute for Electricity and Clean Energy (INEEL), “Wind resource map”, available: *http*:*//sag01*.*iie*.*org*.*mx/sig/*. Accessed: November 2019.

**Fig 9:**
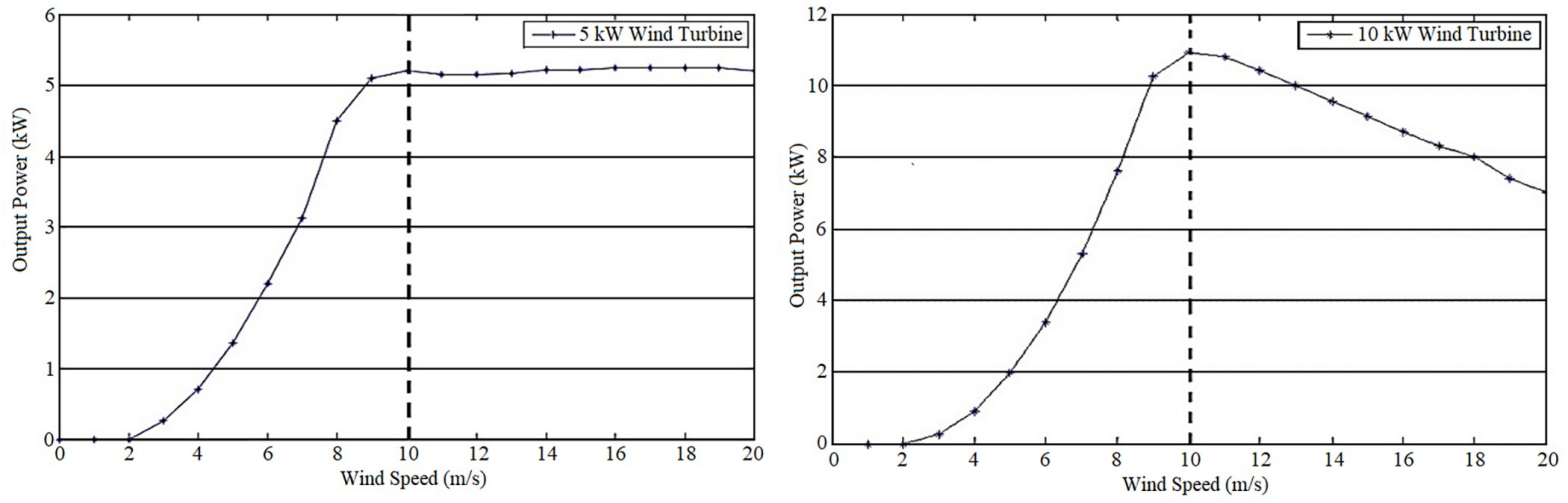
Power curves for the class 4 SSWTs: (a) 5 kW, (b) 10 kW, represented in the nodes Power curve 5 kW SSWT and Power curve 10 kW SSWT, respectively.

On the other hand, we consider 5 and 10 kW class 4 SSWT generators as the technology to be deployed. The corresponding power curves are shown in Fig 9. It is important to notice that the wind speed probability distributions of the cities under consideration go up to 10 m/s. Therefore, we only consider generated power up to 10m/s as shown by the dotted lines in Fig 9.

### BDN for the assessment

**Fig 10:**
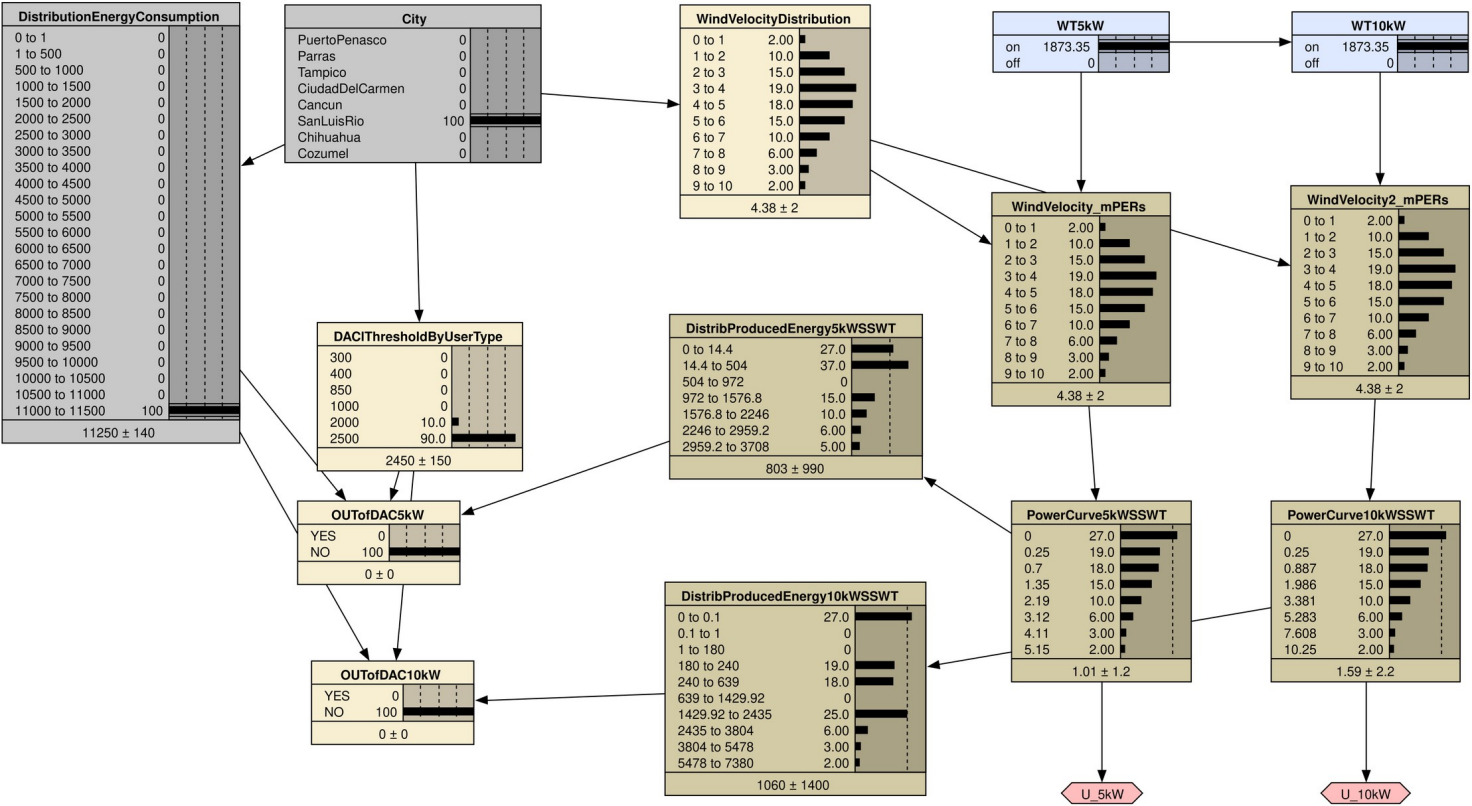
BDN for the assessment of small-scale wind technology deployment in Mexico.

The construction of the BDN for these case studies is done using Netica software [48] and following the methodology outlined in subsection Construction of the decision-making system for Bayesian assessment. The net is shown in Fig 10, for the specific case of the city of Puerto Peñasco. The parent node is “City”. By choosing one city the three main properties defining the scenario at that place are selected: a) the Weibull distribution for the wind speed, b) the maximum limit in energy consumption before the electricity price increases and, c) the discrete probability distribution for the energy consumption of the DAC users. Note that the fact that there is more than one user type at the sites is reflected in the node “DAClimitByUserType”.

The decision nodes “WT5kW” and “WT10kW” select the technology and provide the calculated utility for making this decision which corresponds to the expected energy, *E*_*W*_, produced each month by the SSWT generator and given by Eq (13).

Finally, the nodes “OUTofDAC_5kW” and “OUTofDAC_10kW” calculate the posterior probability that the excess energy *E*_*X*_, given by Eq (11), is smaller than the energy produced by the SSWT generator *E*_*W*_, namely, the *suitability* defined by Eq (12).

### Results of the technical assessment

Table 5 shows a snapshot of the conditions of the decision-making system. The first column corresponds to the mean consumed energy per month, $\bar{E_{C}}$ by DAC users. This quantity corresponds to the mean value of the consumed energy distributions shown in Fig 7. Next, given the DAC threshold per month in the third column, the mean excess energy per month, $\bar{E_{X}}$ is calculated using Eq (11) and reported in the fourth column. The last two columns show the expected energies produced per month, *E*_*W*_, with the 5 and 10 kW SSWT generators calculated using Eq (14).

**Table 5:** Snapshot of the conditions of the decision-making model.

City	Mean consumed energy per month [kWh]	DAC threshold per month [kWh]	Mean excess energy per month [kWh]	Expected energy produced per month with 5 kW SSWT [kWh]	Expected energy produced per month with 10 kW SSWT [kWh]
**Puerto Peñasco**	2710	2000	710	791	1264
**Parras**	459	300	159	874	1416
**Tampico**	2130	850	1280	526	812
**Ciudad del Carmen**	1170	850	320	801	1276
**Cancún**	1540	1000	540	497	752
**San Luis Rio**	3710	2500	1210	726	1147
**Chihuahua**	500	400	100	296	435
**Cozumel**	1290	1000	290	553	846

A deterministic analysis would be based on the comparison of the last three columns to determine the feasibility of the technical exploitation of SSWT technology for a given site. At first sight, one would say that if the energy produced by the SSWT generator is greater than the excess energy,

$$\bar{E_{X}}<E_{w},$$ (18)

it is technically feasible to use SSWT technology to reduce the charge of the electric bills. The cases colored in green are the ones that satisfy this condition, and therefore are the ones where it is technically feasible to exploitof SSWT technology in order to reduce electricity bills. At the same time, the cases colored in red do not satisfy the condition in Eq (13) and one would say that it is not convenient to use SSWT technology to reduce the electricity bills in those cities.

However, the exact amounts of monthly energy produced by the SSWT generators and consumed by the DAC users are not known. Therefore, there is no certainty that Eq (18) is satisfied every month. It follows from this that a probabilistic analysis is more adequate to provide a more realistic assessment of the technical feasibility of using SSWT generators to reduce the electricity bills of the DAC users. As described in the previous section, one way to deal with this problem is with the decision-making model introduced there by calculating the conditional probability of the *suitability*, given the probability distributions functions of the wind speed and the monthly consumed energy.

The *suitability*, defined by Eq (17), for the different sites under consideration is calculated with the BDN model for the assessment of the SSWT technology, Fig 6, and the results are reported in Table 6. The cases colored in green are the ones for which the probability of the *suitability* is closer to or greater than 50%. Therefore, in these cases, it is clear that is convenient to use this technology to reduce electricity bills. At the same time, the cases colored in red are those for which the probability of the *suitability* is smaller than 50%. In other words, in these cities, it is less likely that the use of SSWT generators helps reduce electricity bills.

**Table 6:** Suitability of the technical exploitation of SSWT technology for a given site.

City	*Sutiability* with 5kW SSWT (%)	*Sutiability* with 10kW SSWT (%)
**Puerto Peñasco**	57.1	63.9
**Parras**	77.5	81.0
**Tampico**	29.6	37.7
**Ciudad del Carmen**	61.8	69.8
**Cancún**	43.1	51.5
**San Luis Rio**	59.7	64.2
**Chihuahua**	49.4	64.6
**Cozumel**	63.9	70.6

### Discussion of the results of technical assessment

The soundness of this assessment system lies in the fact that it uses probabilistic reasoning in contrast to deterministic reasoning. By comparing results in Tables 5 and 6 we note three interesting cases:

- Parras with a 10kW SSWT technology corresponds to the case where the difference *E*_*w*_−*E*_*x*_ is the biggest one. Hence, from the deterministic analysis, one would say that it is the best site to implement the technology. Indeed, the *suitability* for this case is the larges and corresponds to 81%. Therefore, the deterministic and the probabilistic reasoning agree that DAC users in this city can use 10 kW SSWT technology to avoid high electricity bills with an 81% probability of success.
- Tampico with a 5 kW SSWT technology corresponds to the case where the difference *E*_*w*_−*E*_*x*_ is negative and the smallest one. Therefore, from the deterministic analysis, one would say that it is the worst city for implementing the technology. Indeed, the probabilistic reasoning performed by the assessment system agrees with this fact since the corresponding *suitability* is the smallest. Indeed, there is only a 29.6% probability that SSWT technology produces enough energy to avoid DAC tariffs.
- San Luis Rio, with 5 and 10 kW SSWT technologies, is a case in which the results from probabilistic reasoning contrast with those from deterministic reasoning. From Table 5, it follows that the difference *E*_*w*_−*E*_*x*_ is negative. Hence, from the deterministic analysis, one would say that it is not worth implementing the technology since the produced energy from the SSWT technology is not enough to bypass the DAC threshold. However, the *suitability* resulting from the probabilistic reasoning is 59.7% and 64.2% for the 5 and 10 kW SSWT, respectively. These results indicate that, in this city, it is technically feasible to use SSWT technology to avoid high electricity bills, opposite to the deterministic analysis.

**Fig 11:**
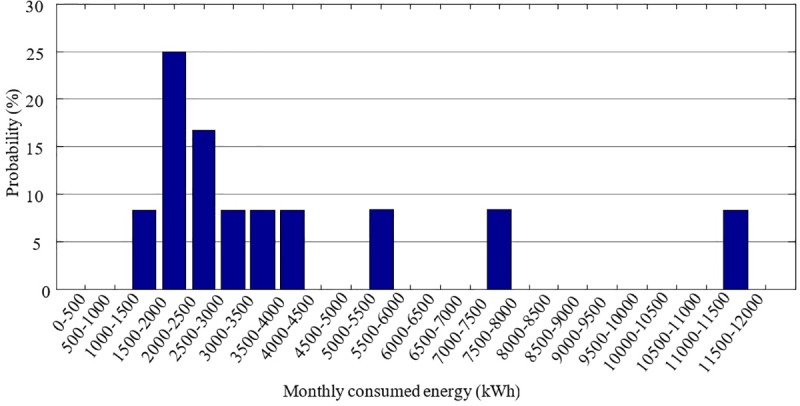
San Luis Rio´s distribution for energy consumption.

**Fig 12:**
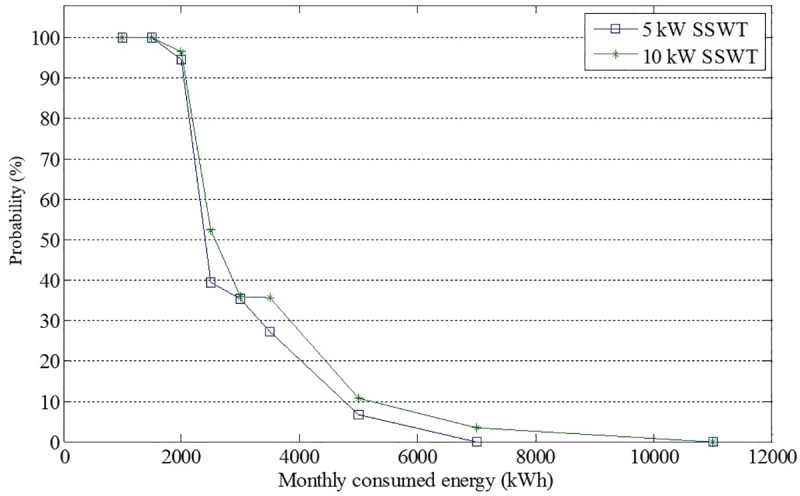
San Luis Rio´s suitability for different values of energy consumption. The blue and green lines correspond to 5 and 10 kW SSWT technologies, respectively.

The reason for this discrepancy is the following. San Luis Rio presents the widest distribution in energy consumption per DAC user as shown in Fig 11. The deterministic reasoning uses the mean consumed energy of the distribution and the expected energy produced by the wind turbine technology to calculate the difference *E*_*w*_−*E*_*x*_. However, the mean consumed energy of the distribution, corresponds to 3710 kWh, as shown in Table 5, and it is not representative of all the possible consumption scenarios indicated in the distribution. At the same time, probabilistic reasoning takes this into account. Fig 12 shows the probability of the suitability for the different possible scenarios for energy consumption for San Luis Rio for 5 and 10kW according to its distribution. As long as the energy consumption per DAC user is less than 3000kWh the probability for the *suitability* could be greater than 50%. However, if the consumed energy per DAC user is larger than 4000 kWh, it is very unlikely that the SSWT technology will be able to avoid large electricity bills. Finally, for consumption above 7000 kWh, the probability to avoid the high cost of electricity is almost zero, or exactly zero, depending on the SSWT generator.

Even though there is an evident congruence between most of the results obtained from both approaches, depending on the energy consumption and wind resource distributions, the results from the probabilistic reasoning may not completely agree with the results from the deterministic energy. Indeed, the narrower the distribution is, the greater the agreement between results from the probabilistic and deterministic reasoning is. On the contrary, with increasing width of the distribution, the divergence between both reasoning arises since a probabilistic treatment becomes necessary.

There are other related works published in the literature. For instance, Rodriguez-Hernandez *et*. *al*. [33] have performed a techno-economic feasibility study of SSWT in the Valley of Mexico Metropolitan Area (VMMA). They consider a Rayleigh probability density function to model wind speed data and use wind speed data samples with 5 and 10 min mean times to estimate the mean annual generated power, using Eq (7), for a variety of SSWT classes. They consider that the generated energy is underestimated because of the sample mean time, and add an additional 30% of energy to compensate for this. Moreover, in an earlier study in 2016, Rodríguez-Hernández *et*. *al*. [49] found the effects of using different mean times in wind power resource assessments and concluded that using larger mean times leads to an underestimation of the energy produced. Our findings avoid this underestimation by using probabilistic reasoning providing a more reliable assessment. Moreover, the techno-economic assessment presented by Rodríguez-Hernández *et*. *al*. in 2019 [33] only takes into consideration the lowest and highest price of kWh of one user type, since they only deal with the VMMA. However, since our approach deals with a variety of cities it considers all available types of users types described in Table 2.

There is also published work published that considers the uncertainty of the assessment of the implementation of SSWTs. In particular, Kwon [50] presents an uncertainty analysis of wind energy potential assessment by using a probabilistic model that considers the variability of wind resources through a Weibull probability density function, as well as an empirical probability model for a power curve. However, with a Monte-Carlo based numerical simulation, he evaluates the expected annual energy production. Once again, even if this methodology started from a probabilistic point of view, taking into account the intrinsic uncertainty of the problem, in the end, the result is an expected quantity, reducing the approach to a deterministic one, which may be useful for other applications.

## Conclusions

In this article, a novel system, based on Bayesian intelligence, is introduced to assess the technical feasibility of SSWT generators to produce enough electric energy to cover the excess demand for intensive energy residential consumers in order for them to avoid high-priced tariffs.

The main components of an assessment of the technical feasibility of SSWT for residential use are the energy produced by the SSWTs and the energy consumed by residential users. Given the fact that both quantities are random, the problem in question contains a degree of uncertainty. Indeed, most of wind technology assessments use average wind speeds and do not acknowledge the fact that wind speed varies significantly during the course of a day or year. However, our findings deal with this lack of certainty by using a probabilistic tool specifically designed to deal with problems with uncertainty, namely, BDNs. As a consequence, instead of obtaining an overestimated or underestimated result of generated energy from SSWTs [51], our Bayesian intelligent system accomplishes a probabilistic assessment. This is done by introducing the concept of *suitability* as a measure of the probability of technical feasibility to use SSWTs to produce the excess energy needed by residential consumers to avoid high priced tariffs.

Our findings contain not only the inherent uncertainty of the wind resource but also the energy demand in the residential sector at every site under consideration. They also take into account the complex kWh price tariff structure in Mexico, as well as the different energy thresholds before a residential user becomes a high domestic user (DAC) for each user type according to CFE. Therefore, three types of complexities are considered by our system to provide an assessment. This is in contrast with a deterministic analysis, where a techno-economical analysis is performed with mean values to provide a crisp value that contains no information about the uncertainty of the problem.

The usefulness and efficacy of the proposed system are demonstrated by its application in a case study testing two types of SSWTs. The probabilistic assessment of technical feasibility is made for eight cities in Mexico with acceptable wind resources to use SSWT technologies and with high residential energy consumption. The results show the suitability of the usage of SSWTs to produce the excess demand for intensive energy residential consumers to avoid high-priced electricity. It turns out, unsurprisingly, that for sites with narrow probability distributions of energy consumption, the probabilistic assessment result is closer to the deterministic assessment result. However, for sites with wide probability distributions of energy consumption, the probabilistic and the deterministic assessments differ, in which case the probabilistic assessment should be preferred since it incorporates the uncertainty of the problem.

The results provided by the assessment system can be utilized by at least three different users: 1) residential consumers can use them to figure out whether SSWT technology is a viable option to get off high-priced electricity tariffs, 2) SSWT providers can use them to identify the best target zones and clients to sell their products and, 3) the electric utility company CFE can use them to improve the distribution of the electric energy, for example by routing the excess energy to industrial consumers instead of residential consumers.

There are several improvements that can be done to the system in order to obtain a more accurate assessment. For instance, in the present study, we used the annual Weibull probability distribution for the wind speed to calculate the probability distribution for the produced energy. This is a weakness of the system as, in most sites, wind speeds present a greater variation between seasons; therefore, it would be desirable to include a season, or monthly, Weibull probability density function for wind speed. Additionally, it would be better if more data for energy consumption could be used to construct the energy consumption probabilistic distribution function. In this work, we only used monthly data corresponding to one year since we could not obtain more. Moreover, if data from several years were available it would be interesting to include a monthly demand profile for energy consumption since it changes drastically from summer to winter. At the same time, one can identify some limitations of the system. First, there is no way to claim that the structure of the BDN model is unique: all the processes involved in the construction of the BDN model rely on the KEBN as explained in the Knowledge Engineering with BNs subsection. Second, the results strongly depend on the data; therefore, one must count with reliable data to trust the results. Finally, there are different obstacles in urban areas, such as buildings and trees, which may change the wind flow profile and may decrease the energy produced by the SSWT. In this study, we have considered laminar flow, neglecting any kind of turbulence created due to the city elements configurations.

This work is planned to be extended in several directions. First, we will look for a bigger set of wind data to incorporate the seasonal or monthly wind speed probability distributions. Likewise, we have to look for a way to introduce the possible turbulence effects produced by obstacles due to urban orography. Moreover, the economic aspect will be included in the assessment system in order to obtain a techno-economical assessment for the implementation of the SSWT technology, via additional utility nodes representing in more detail the energy pricing structure of the kWh. Next, hybrid distributed generation, with low carbon footprint systems, will be considered by integrating other technologies, such as photovoltaic and electric storage devices, since it is believed that small-scale hybrid renewable distributed generation can provide the best solutions to produce clean and affordable energy for residential use with several other valuable benefits. In this way, the assessment system could be able to provide a complete techno-economical assessment for hybrid renewable technologies to boost the implementation of clean energy.

Currently, Mexico is committed to meet the goals established in the Law on the Use of Renewable Energies and Financing of the Energy Transition, LAERFTE [52], and the Law of Climate Change [53], so that by 2024 a minimum of 35% of the electricity comes from renewable energy sources. To carry out the energy transition process and to comply with the aforementioned goal, the Law of Energy Transition [54], relies on strategies, programs and public policies that boost the use of clean energies. At this time, there are obstacles for the use energy from renewable sources—and in particular wind energy—in Mexico. However, more detailed feasibility studies are still necessary to fully exploit these energy sources. Specifically, this work is related to identifying zones of high potential for the use of 5 and 10 kW SSWTs, which are the only ones currently manufactured in Mexico, in the residential sector.

## Supporting information

S1 Table Monthly average energy consumption (in kWh) per DAC user given in 2018. (DOCX)

S1 Fig Normalized wind speed distributions for Puerto Peñasco, Parras, Tampico and Ciudad del Carmen. These data were obtained from National Institute for Electricity and Clean Energy (INEEL), “Wind resource map”, available at http://sag01.iie.org.mx/sig/. Accessed in November 2019.
(TIF)

S2 Fig Normalized wind speed distributions for Cancun, San Luis Rio, Chihuahua, and Cozumel. These data were obtained from National Institute for Electricity and Clean Energy (INEEL), “Wind resource map”, available at http://sag01.iie.org.mx/sig/. Accessed in November 2019.
(TIF)
