# Supplementary material for: Technical assessment of small-scale wind power for residential use in Mexico: A Bayesian intelligence approach
Source: PLoS One. 2020 Mar 12;15(3):e0230122. doi: 10.1371/journal.pone.0230122 (PMC7067485; doi:10.1371/journal.pone.0230122)
Supplement: S1 Table — (DOCX) [file pone.0230122.s001.docx]

**S1 Table. Monthly average energy consumption (in kWh) per DAC user given in 2018.**

| **City** | **January** | **February** | **March** | **April** | **May** | **June** | **July** | **August** | **Sept** | **October** | **Nov/Dec** |
| --- | --- | --- | --- | --- | --- | --- | --- | --- | --- | --- | --- |
| Pto. Peñasco | 1222.5 | 1026 | 1343.5 | 1362.5 | 1762.7 | 2174.2 | 4116.5 | 5152.8 | 5368.7 | 4029.6 | 4100 |
| Parras | 575.9 | 544.7 | 385.7 | 416.1 | 486.2 | 538.4 | 519.5 | 548.9 | 460.8 | 440.6 | 826 |
| Tampico | 925.6 | 1625.1 | 1010.4 | 1742.2 | 1478.7 | 3900.3 | 2351.5 | 4528.4 | 2375.1 | 4574.1 | 1800 |
| Cd. Carmen | 648.4 | 845.8 | 681.5 | 1286.7 | 937.9 | 1601.8 | 1131.1 | 1560.4 | 1036.2 | 1471.9 | 2183 |
| Cancun | 1199.9 | 1020 | 1350.3 | 1077.9 | 2496.2 | 1046.5 | 1987.4 | 1519.3 | 2456.9 | 1242.4 | 2845 |
| San Luis Río | 1666.5 | 2203.5 | 1463.8 | 1592.5 | 2482 | 2946.5 | 3797 | 7059.5 | 11369.2 | 5029.1 | 4743 |
| Chihuahua | 473.3 | 629.8 | 424.9 | 445.6 | 381.9 | 685.3 | 607.9 | 815.8 | 572 | 555 | 895 |
| Cozumel | 603.5 | 1645.3 | 563.2 | 1893.2 | 741.2 | 1540.3 | 895.1 | 2186 | 1038.7 | 1622.8 | 2309 |

These data were obtained from Federal Electricity Commission (CFE), “Users and electricity consumption by municipality”, available at <https://datos.gob.mx/busca/dataset/usuarios-y-consumo-de-electricidad-por-municipio-a-partir-de-2018> and accessed in November 2019. Note that for the last two months of the year there is only published data for the bimester, then through an average calculation, we estimate the consumed energy in November and December to obtain the discrete probability distribution of the average monthly consumed energy by the DAC users shown in Fig. 8.
