# Supplementary figures and images for: Technical assessment of small-scale wind power for residential use in Mexico: A Bayesian intelligence approach
Source: PLoS One. 2020 Mar 12;15(3):e0230122. doi: 10.1371/journal.pone.0230122 (PMC7067485; doi:10.1371/journal.pone.0230122)

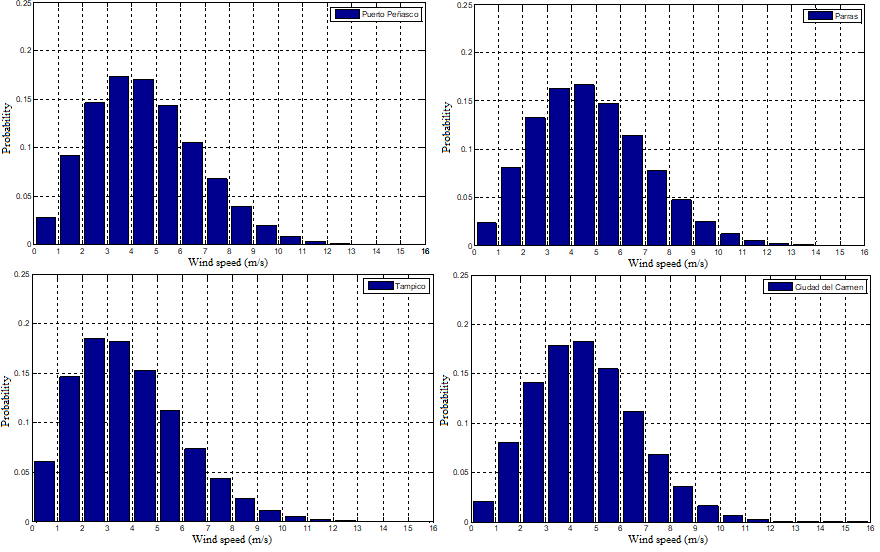

Supplement: S1 Fig — These data were obtained from National Institute for Electricity and Clean Energy (INEEL), “Wind resource map”, available at http://sag01.iie.org.mx/sig/. Accessed in November 2019. (TIF) [file pone.0230122.s002.tif]

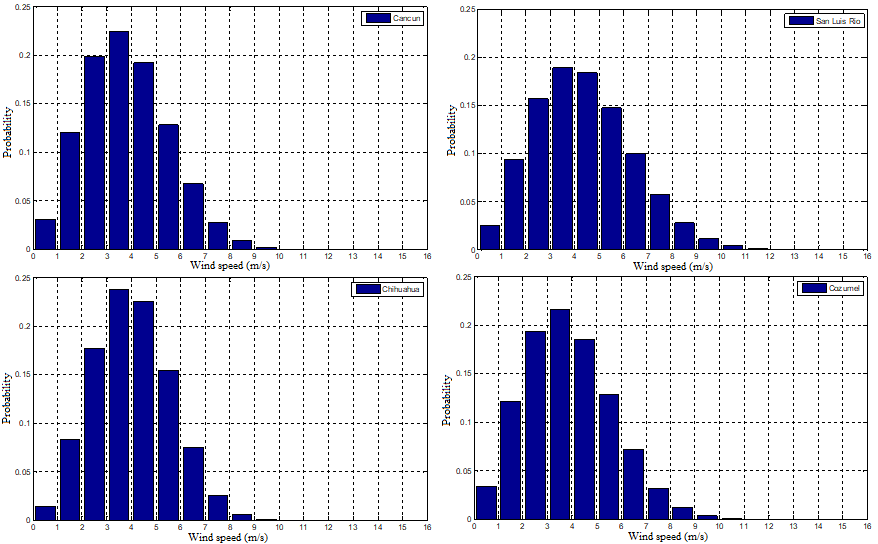

Supplement: S2 Fig — These data were obtained from National Institute for Electricity and Clean Energy (INEEL), “Wind resource map”, available at http://sag01.iie.org.mx/sig/. Accessed in November 2019. (TIF) [file pone.0230122.s003.tif]
